# Clinical Impact of HRCT UIP Pattern Among Patients With Idiopathic Pulmonary Fibrosis Undergoing Antifibrotic Treatment: A Multicenter Retrospective Observational Study

**DOI:** 10.1155/carj/3604892

**Published:** 2026-09-10

**Authors:** Satoshi Nagaoka, Yu Hara, Hiroaki Fujii, Kota Murohashi, Ryo Nagasawa, Yoichi Tagami, Yusuke Saigusa, Tatsuji Enomoto, Yutaka Matsumoto, Makoto Masuda, Nobuyuki Horita, Masaki Yamamoto, Nobuaki Kobayashi, Takashi Ogura, Takeshi Kaneko, Makoto Kudo

**Affiliations:** ^1^ Department of Pulmonology, Yokohama City University Graduate School of Medicine, 3-9 Fukuura Kanazawa-ku, Yokohama 236-0004, Kanagawa, Japan, yokohama-cu.ac.jp; ^2^ Department of Pulmonology, Yokohama Minami Kyousai Hospital, 1-21-1 Mutsuurahigasi Kanazawa-ku, Yokohama 236-0037, Kanagawa, Japan, minamikyousai.jp; ^3^ Department of Respiratory Medicine, Kanagawa Cardiovascular and Respiratory Center, 6-16-1 Tomioka-higashi Kanazawa-ku, Yokohama 236-0051, Kanagawa, Japan; ^4^ Department of Biostatistics, Yokohama City University Graduate School of Medicine, 3-9 Fukuura Kanazawa, Yokohama 236-0004, Japan, yokohama-cu.ac.jp; ^5^ Department of Respiratory Medicine, Ofuna Chuo Hospital, 6-2-24 Ofuna, Kamakura 247-0056, Kanagawa, Japan, ofunachuohp.net; ^6^ Department of Respiratory Medicine, Yamato Municipal Hospital, 8-3-6 Fukami-nishi, Yamato 242-8602, Kanagawa, Japan; ^7^ Department of Respiratory Medicine, Fujisawa City Hospital, 2-6-1 Fujisawa, Fujisawa 251-8550, Kanagawa, Japan; ^8^ Respiratory Disease Center, Yokohama City University Medical Center, 4-57 Urafune-cho Minami-ku, Yokohama 232-0024, Kanagawa, Japan, yokohama-cu.ac.jp

**Keywords:** antifibrotic treatment, idiopathic pulmonary fibrosis, nintedanib, pirfenidone, usual interstitial pneumonia

## Abstract

**Background:**

Idiopathic pulmonary fibrosis (IPF) is a progressive fibrosing interstitial lung disease with a poor prognosis. Honeycombing on high‐resolution computed tomography (HRCT) is a defining feature of the usual interstitial pneumonia (UIP) pattern and has traditionally been considered a marker of advanced disease. However, in the era of antifibrotic treatment (AFT), the prognostic significance of the UIP pattern in IPF remains uncertain. This study aimed to evaluate the association between HRCT UIP pattern and long‐term prognosis in patients with IPF receiving AFT.

**Methods:**

This retrospective multicenter study investigated consecutive patients with IPF who initiated AFT between 2009 and 2019 at seven institutions in Japan. HRCT patterns were classified as UIP or non‐UIP by multidisciplinary assessment. The primary outcome was IPF‐related events, defined as IPF‐related death or first acute exacerbation (AE) after initiating AFT. Event‐free survival was analyzed using Kaplan–Meier methods with stratification by gender–age–physiology (GAP) stage. Propensity score matching (PSM) was performed to adjust for age, sex, GAP score, and antifibrotic agent.

**Results:**

Of the 139 patients included, 99 had a UIP pattern, and 40 had a non‐UIP pattern. In the overall cohort, event‐free survival did not differ significantly. Stratified analyses showed no significant survival difference between UIP and non‐UIP patterns among GAP Stage I patients (median survival time [MST]: 2050 vs. 1104 days; *p* = 0.90). Similarly, among patients with GAP Stages II–III, MST did not differ significantly. After PSM, 34 matched pairs were analyzed, and no significant differences in event‐free survival were observed between UIP and non‐UIP patterns.

**Conclusions:**

In patients with IPF treated with AFT, the radiological UIP pattern on HRCT was not independently associated with long‐term prognosis. Multidimensional risk assessment incorporating physiological impairment and disease behavior may provide more accurate prognostic stratification in treated UIP populations.

## 1. Introduction

Idiopathic pulmonary fibrosis (IPF) is a chronic, progressive fibrosing interstitial lung disease (ILD) of unknown etiology that predominantly affects older adults. IPF is characterized by irreversible architectural distortion of the lung parenchyma due to excessive deposition of extracellular matrix, ultimately leading to impaired gas exchange and respiratory failure [1–3]. Despite advances in diagnostic strategies and therapeutic options, IPF remains a fatal disease, with a reported median survival of approximately 2–5 years after diagnosis. Disease progression, chronic respiratory failure, and acute exacerbation (AE) represent the major causes of mortality [4].

Over the past decade, antifibrotic treatment (AFT), including nintedanib and pirfenidone, has been globally approved and incorporated into standard clinical practice for IPF. These agents have been consistently shown to slow the rate of decline in forced vital capacity and to reduce the incidence of AEs, and accumulating evidence suggests that they may also contribute to improved long‐term survival [5, 6]. As a result, the clinical course of IPF has been substantially modified, shifting the disease paradigm from an inevitably rapid decline to a more heterogeneous and, in some cases, slowly progressive trajectory under treatment. Nevertheless, the clinical course and rate of progression of IPF vary widely among patients. Numerous prognostic factors have been proposed, including clinical symptoms, physiological parameters, radiological and pathological features, and circulating or bronchoalveolar lavage fluid biomarkers [1, 2]. However, no single biomarker has been definitively established as a reliable predictor of long‐term outcomes in routine clinical practice. Among the radiological findings, honeycombing on high‐resolution computed tomography (HRCT) represents the hallmark feature of the usual interstitial pneumonia (UIP) pattern and has traditionally been regarded as an indicator of advanced and irreversible fibrosis. Honeycombing is defined as clustered cystic airspaces, typically 3–10 mm in diameter but occasionally larger, with thick and well‐defined walls, and is commonly accompanied by reticular abnormalities and traction bronchiectasis [7].

Honeycombing is not specific to IPF and can be observed in various fibrotic ILDs, including chronic hypersensitivity pneumonitis (CHP), connective tissue disease (CTD)–associated ILD, and unclassifiable ILD (UC‐ILD) [8]. In current clinical guidelines, the presence of a UIP pattern on HRCT plays a pivotal role in the diagnosis of IPF and often serves as a key determinant for initiating AFT. Historically, honeycombing has also been interpreted as a surrogate marker of disease severity and poor prognosis. However, emerging evidence suggests that the prognostic significance of honeycombing may differ according to the underlying ILD subtype. Indeed, a large multicenter study from the United States involving 1330 patients with fibrotic ILDs (including CHP, CTD‐ILD, IPF, and UC‐ILD) demonstrated honeycombing in 63% of cases. The presence of honeycombing was associated with worse survival in patients with CHP, CTD‐ILD, and UC‐ILD, but was not significantly associated with long‐term prognosis in patients with IPF [8]. These findings raise important questions regarding the conventional assumption that honeycombing uniformly portends a poor outcome in IPF, particularly in the current era of AFT.

Against this background, the prognostic value of the UIP pattern (specifically, the presence or absence of honeycombing on HRCT) among patients with IPF receiving AFT remains incompletely understood. The present study aimed to investigate whether the presence of a UIP pattern on HRCT is associated with long‐term prognosis among patients with IPF treated using antifibrotic agents.

## 2. Materials and Methods

### 2.1. Study Design and Patient Enrollment

This retrospective observational study included consecutive patients with IPF who initiated AFT between 2009 and 2019 at the following institutions in Japan: Yokohama City University Hospital, Yokohama City University Medical Center, Kanagawa Cardiovascular and Respiratory Center, Ofuna Chuo Hospital, Fujisawa City Hospital, Yokohama Minami Kyousai Hospital, and Yamato Municipal Hospital. IPF was diagnosed according to the official diagnostic criteria jointly published by the American Thoracic Society, European Respiratory Society, Japanese Respiratory Society, and Latin American Thoracic Society (ATS/ERS/JRS/ALAT) [2]. Diagnostic classification was established through multidisciplinary assessment at each participating institution according to contemporaneous guideline criteria. The diagnosis of CT‐ILD was confirmed by physical findings, serological testing, and HRCT findings that were consistent with ILD. Histopathological confirmation was not systematically available because of the retrospective multicenter design and because surgical lung biopsy was not routinely performed in all patients. The decision to initiate AFT was made at the discretion of the attending physician at each participating center.

### 2.2. Data Collection

The following data were retrospectively collected from medical records at the time of AFT initiation: age, sex, smoking history, pulmonary function test (PFT) results, HRCT pattern (classified as UIP, probable UIP, or indeterminate for UIP), gender–age–physiology (GAP) score, and the type of antifibrotic agent (nintedanib or pirfenidone) administered [2, 9]. GAP score was calculated according to the original GAP model using sex, age, percentage predicted forced vital capacity (%FVC), and percentage predicted diffusing capacity of the lungs for carbon monoxide (%DLco), and patients were categorized into GAP Stages I–III as previously described [9]. The interval between PFT and HRCT acquisition was within 6 months. In addition, we evaluated treatment duration, dosage of antifibrotic agents, and disease outcomes. Disease outcomes were defined as IPF‐related events, including IPF‐related death due to respiratory failure or the first occurrence of AE following the initiation of AFT. The observation period for survival analysis started on the date of the PFT used for GAP score calculation. Patients were followed until the occurrence of IPF‐related death or the first AE. Patients were continuously followed even after discontinuation or completion of AFT. AE was defined as an acute respiratory deterioration requiring steroid pulse therapy, characterized by new or worsening dyspnea, hypoxemia, or severely impaired gas exchange, along with new bilateral ground‐glass opacities and/or consolidation on HRCT, superimposed on a background pattern consistent with IPF, not fully explained by cardiac failure or fluid overload [4].

HRCT patterns (UIP vs. non‐UIP, including probable UIP and indeterminate for UIP) were independently assessed using baseline HRCT images obtained at the initiation of AFT by experienced chest physicians and radiologists. HRCT patterns were classified according to the 2018 ATS/ERS/JRS/ALAT guideline as UIP, probable UIP, or indeterminate for UIP [2]. A UIP pattern was defined by the presence of subpleural and basal‐predominant fibrosis with honeycombing, with or without peripheral traction bronchiectasis or bronchiolectasis. Probable UIP was defined as subpleural and basal‐predominant reticular abnormalities accompanied by peripheral traction bronchiectasis or bronchiolectasis in the absence of honeycombing. Indeterminate for UIP was assigned when HRCT demonstrated fibrotic abnormalities that did not fulfill the criteria for UIP or probable UIP and did not suggest an alternative diagnosis. This category included cases with subtle reticulation, mild ground‐glass opacities, or early fibrotic changes insufficient for classification as UIP or probable UIP. Also, particular attention was paid to distinguishing honeycombing from traction bronchiolectasis, paraseptal emphysema, and subpleural cystic changes. Honeycombing was defined as clustered subpleural cystic airspaces with well‐defined walls, and in cases of disagreement, a consensus was reached through multidisciplinary discussion among the reviewers [2].

### 2.3. Statistical Analysis

Categorical variables were compared using Fisher’s exact test, while continuous variables were analyzed using the Wilcoxon rank‐sum test. Kaplan–Meier survival analysis and Cox proportional hazards models were used to compare median survival times (MSTs) and hazard ratios (HRs) between the UIP and non‐UIP pattern groups. Propensity score matching (PSM) was conducted to adjust for potential confounders, including age, sex, GAP score, and type of antifibrotic agent. Variables were matched using a caliper width such that standardized differences remained below 0.25, in accordance with commonly accepted criteria. Age, sex, and GAP score were selected based on the reported prognostic relevance in IPF, while the type of antifibrotic agent was included as the key treatment‐related variable. Facility and attending physician were not included as matching variables, as doing so would have significantly limited the number of matched pairs. As a sensitivity analysis, inverse probability weighting (IPW) based on propensity scores was additionally performed to further account for potential baseline imbalances between the UIP and non‐UIP groups. Propensity scores were estimated using a logistic regression model including age, %FVC, and %DLco, which represent the principal physiological components of the GAP model and are established prognostic factors in IPF. Stabilized inverse probability weights were applied to generate a weighted pseudo‐population, and covariate balance was assessed using standardized mean differences (SMDs), with values < 0.20 considered acceptable. Weighted Kaplan–Meier survival analyses and Cox proportional hazards models were subsequently performed. All statistical analyses were performed using JMP Pro (Version 18.0.2; JMP Statistical Discovery LLC, Cary, NC, USA) and R (Version 4.5.0; R Foundation for Statistical Computing, Vienna, Austria). A two‐sided *p* value < 0.05 was considered statistically significant.

## 3. Results

### 3.1. Patient Characteristics

**FIGURE 1:**
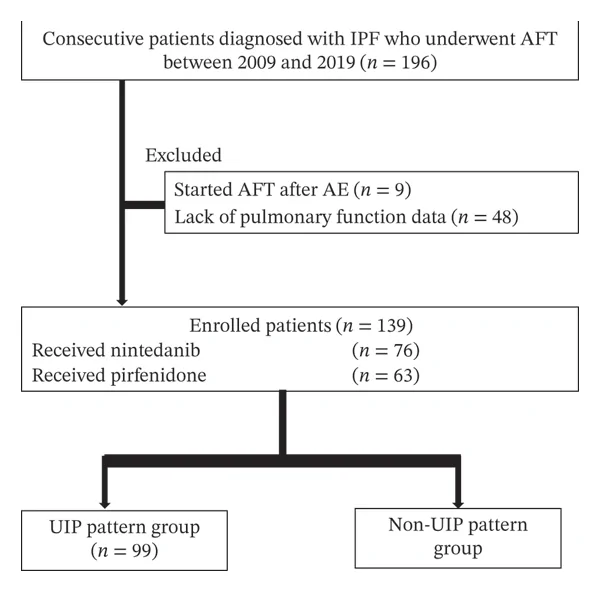
Flowchart of participant selection.

**FIGURE 2:**
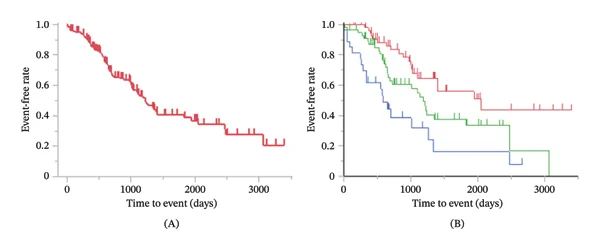
Kaplan–Meier survival curve for time to death or AE of all patients according to GAP stage for all patients (A) or according to GAP stage (B). MST among all patients was 1225 days (A). GAP stage was I in 56 patients, II in 56 patients, and III in 27 patients. Red, green, and blue lines indicate GAP Stages I, II, and III, respectively. MST was 2050 days for Stage I, 1188 days for Stage II, and 587 days for Stage III. Comparison by log‐rank test revealed significant differences among groups. Abbreviations: AE, acute exacerbation; GAP, gender–age–physiology; MST, median survival time.

A flowchart of the participant selection process is shown in Figure 1. Among 196 consecutive patients diagnosed with IPF who underwent AFT between June 2009 and June 2019, the 57 patients without pulmonary function data (including the diffusing capacity of the lungs for carbon monoxide [DLco]) or with initiation of AFT after AE were excluded. As a result, 139 patients were evaluated, classified into the UIP pattern group (*n* = 99, 71%) and the non‐UIP pattern group (*n* = 40, 29%). Non‐UIP patterns included probable UIP (*n* = 12) and indeterminate for UIP (*n* = 28). Nintedanib was used in 76 patients and pirfenidone in 63 patients. As shown in Table 1, no significant differences were seen in baseline characteristics, including GAP score, types of antifibrotic drugs, and mean dosage of AFT, with the exception of sex, smoking history, and duration of pirfenidone administration. GAP stage was Stage I in 56 patients, Stage II in 56, and Stage III in 27. Figure 2 shows Kaplan–Meier survival curves for IPF‐related events according to GAP stage. Comparison by log‐rank test revealed a significant difference among groups (*p* < 0.01).

**TABLE 1:** Patient characteristics.

Variable	Total (*N* = 139)	UIP pattern (*n* = 99)	Non‐UIP pattern (*n* = 40)	*p* value
Age, years	74 (8.1)	74 (6.3)	72 (10.6)	< 0.01
Sex, male/female (%)	107/32 (77/23)	82/17 (83/17)	25/15 (63/37)	0.01
Never‐smoker, *n* (%)	38 (27)	22 (22)	16 (40)	0.03
BMI, kg/m^2^	23.7 (3.4)	23.7 (3.4)	23.8 (3.4)	0.99
Pulmonary function				
%FVC	76.6 (19.46)	76.7 (20.9)	72.5 (15.3)	0.42
%DLco	59.7 (20.7)	59.4 (21.3)	61.9 (19.3)	0.38
HRCT pattern				
Probable UIP (%)			12 (30)	
Indeterminate for UIP (%)			28 (70)	
Treatment options				
Antifibrotic treatment				
Nintedanib/pirfenidone (%)	76/63 (55/45)	56/43 (57/43)	20/20 (50/50)	0.48
Mean dose of nintedanib (mg)	277.3	287.8	247.1	0.59
Mean dose of pirfenidone (mg)	1126.7	1053.7	1284.2	0.06
Dosing period of nintedanib (days)	299.5	224	431.5	0.16
Dosing period of pirfenidone (days)	400	476	236	0.05
Anti‐inflammatory treatment				
Corticosteroid	27 (19)	20 (20)	7 (18)	0.72
Immunosuppressants	11 (8)	6 (6)	5 (13)	0.20
GAP score	5	4	5	0.35
Stage I (score 0–3)	56	40	16	
Stage II (score 4–7)	56	37	19	
Stage III (score 8–14)	27	22	5	
Outcomes				
AE (%)	49 (35)	37 (37)	12 (30)	0.41
Death (%)	56 (40)	41 (41)	15 (38)	0.67

*Note:* Data are presented as median and interquartile range or number and percentage. %DLco, percentage predicted diffusion capacity of lungs for carbon monoxide; %FVC, percentage predicted forced vital capacity.

Abbreviations: GAP, gender–age–physiology; HRCT, high‐resolution computed tomography; IPF, idiopathic pulmonary fibrosis; UIP, usual interstitial pneumonia.

### 3.2. Comparison of Prognosis Between UIP and Non‐UIP

**FIGURE 3:**
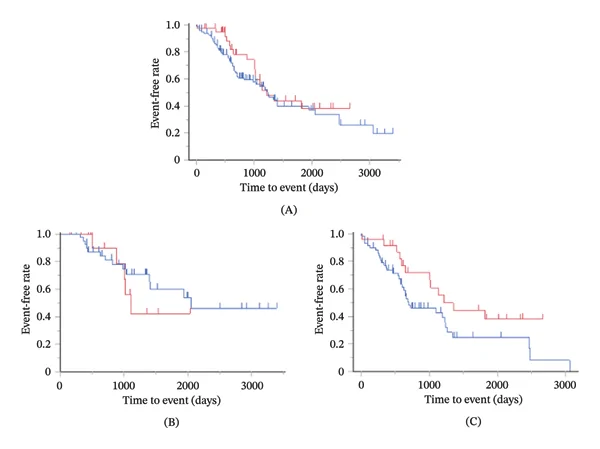
Kaplan–Meier survival curve for time to death or AE among UIP pattern and non‐UIP pattern groups for all patients (A) and patients with GAP Stage I (B) or Stage II or III (C). Blue and red lines indicate the UIP and non‐UIP pattern groups, respectively. In the overall cohort, event‐free survival did not differ significantly between patients with UIP and those with non‐UIP patterns (A). Blue and red lines indicate the UIP and non‐UIP pattern groups, respectively. When stratified according to GAP stage, no significant difference in event‐free survival was observed between UIP and non‐UIP patterns in either GAP Stage I or Stage II + III patients. Abbreviations: AE, acute exacerbation; GAP, gender–age–physiology; UIP, usual interstitial pneumonia.

Kaplan–Meier survival analyses comparing patients with UIP and non‐UIP patterns are shown in Figure 3. In the overall cohort, event‐free survival did not differ significantly between patients with UIP and those with non‐UIP patterns (Figure 3A). When stratified according to GAP stage, no significant difference in survival was observed between UIP and non‐UIP patterns among GAP Stage I patients (UIP, *n* = 40; non‐UIP, *n* = 16; *p* = 0.90; Figure 3B). The MST in GAP Stage I was 2050 days for the UIP group and 1104 days for the non‐UIP group. Similarly, among patients with advanced disease (GAP Stages II and III), survival did not differ significantly between UIP (*n* = 59) and non‐UIP (*n* = 24) patterns, although a trend toward poorer outcomes in the UIP group was observed (*p* = 0.07; Figure 3C). MST was 701 days in the UIP group and 1218 days in the non‐UIP group. Overall, these findings indicate that a radiological UIP pattern was not independently associated with long‐term prognosis when patients were stratified by GAP stage.

### 3.3. Comparison of Prognosis Between UIP and Non‐UIP After PSM

**FIGURE 4:**
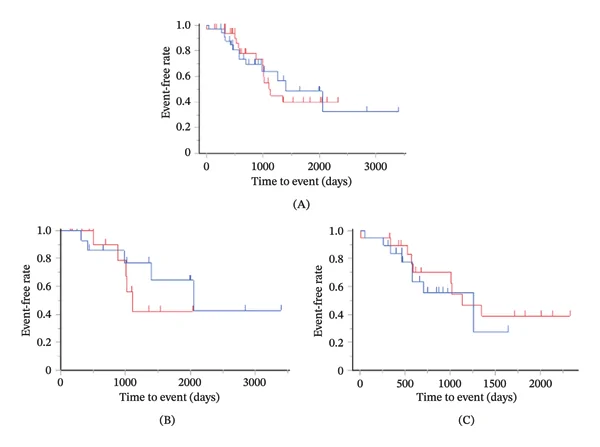
Kaplan–Meier survival curve for time to death or AE among UIP or non‐UIP pattern groups for all patients (A) and patients with GAP Stage I (B) or Stages II or III (C) after PSM: Blue and red lines indicate the UIP and non‐UIP pattern groups, respectively. In the overall cohort, event‐free survival did not differ significantly between patients with UIP and non‐UIP patterns (A). Blue and red lines indicate the UIP and non‐UIP pattern groups, respectively. When stratified according to GAP stage, no significant difference in event‐free survival was observed between UIP and non‐UIP patterns in either GAP Stage I or Stage II + III patients. Abbreviations: AE, acute exacerbation; GAP, gender–age–physiology; UIP, usual interstitial pneumonia.

Baseline characteristics after PSM are summarized in Table 2. After PSM, 68 patients were included, comprising 34 patients with a UIP pattern and 34 with a non‐UIP pattern. No significant differences were evident between the two groups in terms of age, sex distribution, antifibrotic agent use (nintedanib or pirfenidone), dosing, treatment duration, or modified GAP score. The distribution of GAP stages was also well balanced between the UIP and non‐UIP groups, indicating adequate covariate balance after PSM. Kaplan–Meier analyses of event‐free survival in the PSM cohort are shown in Figure 4. In the overall matched population, event‐free survival did not differ significantly between patients with UIP and non‐UIP patterns (Figure 4A).

**TABLE 2:** Patient characteristics after PSM.

Variable	Total (*N* = 68)	UIP pattern (*n* = 34)	Non‐UIP pattern (*n* = 34)	*p* value
Age, years	73.5 (7.4)	73 (7.1)	74 (7.9)	0.80
Sex, male/female (%)	49/19 (72/28)	25/9 (74/26)	24/10 (71/29)	0.79
AFT				
Nintedanib/pirfenidone (%)	39/29 (57/43)	20/14 (59/41)	19/15 (56/44)	0.81
Mean dose of nintedanib (mg)	248.6	247.1	250.0	1.00
Mean dose of pirfenidone (mg)	1179.3	1100.0	1253.3	0.37
Dosing period of nintedanib (mg)	427.1	343.3	510.7	0.25
Dosing period of pirfenidone (mg)	485.6	587.3	390.7	0.14
GAP score	6	6	6	0.51
Stage I (score 0–3)	27	12	15	
Stage II (score 4–7)	28	13	15	
Stage III (score 8–14)	13	9	4	

*Note:* Data are presented as median and interquartile range or number and percentage. %DLco, percentage predicted diffusion capacity of lungs for carbon monoxide; %FVC, percentage predicted forced vital capacity.

Abbreviations: GAP, gender–age–physiology; HRCT, high‐resolution computed tomography; UIP, usual interstitial pneumonia.

When stratified by GAP stage, no significant difference in survival was observed between UIP and non‐UIP patterns among patients with GAP Stage I disease (UIP, *n* = 15; non‐UIP, *n* = 15; *p* = 0.71; Figure 4B). MST was 2050 days in the UIP group and 1104 days in the non‐UIP group. Similarly, among patients with advanced disease (GAP Stages II or III), survival did not differ significantly between UIP (*n* = 19) and non‐UIP (*n* = 19) groups (*p* = 0.52; Figure 4C). MST was 1258 days in the UIP group and 1132 days in the non‐UIP group. These results indicate that, even after adjusting for baseline clinical characteristics using PSM, a radiological UIP pattern was not associated with long‐term prognosis.

### 3.4. Comparison of Prognosis Between UIP and Non‐UIP After IPW

As a sensitivity analysis, IPW was applied using age, %FVC, and %DLco as covariates. Covariate balance was achieved after weighting, with all SMDs below 0.20 (Supporting Table 1). The results of the IPW‐adjusted analyses were consistent with those of the primary and propensity score–matched analyses. No significant difference in event‐free survival was observed between the UIP and non‐UIP groups in the weighted Kaplan–Meier analysis, and radiological UIP pattern was not significantly associated with outcomes in the weighted Cox proportional hazards model (HR 1.28, 95% CI 0.69–2.37, *p* = 0.44; Supporting Figure 1 and Supporting Table 2).

## 4. Discussion

In this multicenter retrospective study, we evaluated the prognostic significance of radiological UIP characterized by honeycombing on HRCT among patients with IPF undergoing AFT. Honeycombing has long been considered a radiological marker of advanced disease and poor prognosis in fibrotic ILDs. However, the prognostic relevance of this finding in IPF under AFT has remained uncertain. Our study demonstrated that the presence of a UIP pattern was not associated with long‐term IPF‐related outcomes, including mortality and AEs, either in the overall cohort or after stratification by disease severity using GAP stage. Importantly, this lack of association persisted even after adjustment for major prognostic factors through PSM. Furthermore, the absence of a prognostic difference between UIP and non‐UIP patterns was confirmed in IPW‐adjusted analyses, supporting the robustness of the findings across different approaches for confounding adjustment. These findings suggest that the diagnostic and prognostic significance of honeycombing may diverge in the current antifibrotic era.

Honeycombing is a defining feature of the definite UIP pattern on HRCT, and is widely regarded as a radiological marker of advanced and irreversible fibrosis in IPF. Consistent with this concept, several cohort studies have demonstrated that patients with definite UIP or CT honeycombing exhibit poorer survival or higher risk of death or lung transplantation compared with those without honeycombing [10–12], supporting the interpretation that honeycombing reflects a greater cumulative fibrotic burden and more advanced disease stage. For example, Salisbury et al. demonstrated that, among patients with IPF, the presence of CT honeycombing was associated with a significantly increased risk of death or lung transplantation (HR 2.04, 95% CI 1.23–3.41), underscoring the adverse prognostic implications of radiological honeycombing in routine clinical practice [10]. Similarly, several analyses comparing definite UIP with probable or possible UIP patterns have reported longer transplant‐free survival in patients lacking honeycombing, further supporting the concept that progression to a honeycomb‐dominant phenotype reflects a transition to a more advanced and prognostically unfavorable stage of IPF [11, 12]. Collectively, these data support the widely held view that UIP with honeycombing represents a high‐risk fibrotic phenotype at the population level, consistent with its interpretation as a marker of advanced irreversible fibrosis.

However, the absence of honeycombing should not necessarily be interpreted as representing an earlier stage of IPF. Current evidence suggests that probable UIP on HRCT frequently corresponds to histopathological UIP and may be observed across a wide spectrum of disease severity. Although honeycombing has traditionally been regarded as a marker of advanced fibrosis, fibrotic remodeling does not invariably progress toward extensive honeycomb formation. In some patients, fibrosis may evolve predominantly as reticulation and traction bronchiectasis, whereas others develop marked honeycombing despite similar physiological impairment. Therefore, the distinction between UIP and probable UIP patterns may reflect differences in the morphological expression of fibrosis rather than a simple temporal progression from early to advanced disease. This concept provides a plausible explanation for the comparable long‐term outcomes observed between radiological categories in the present cohort. Consistent with this interpretation, accumulating evidence indicates that the prognostic impact of UIP categorization or the presence of honeycombing becomes less apparent when analyses are restricted to patients with IPF. Adegunsoye et al. demonstrated that although CT honeycombing was associated with increased mortality across a broad spectrum of fibrotic ILDs, mortality did not differ between IPF patients with and without honeycombing after multivariable adjustment [8]. Similarly, Le Rouzic et al. reported that baseline HRCT pattern, including UIP versus non‐UIP classification, was not independently associated with long‐term survival in IPF, whereas physiological impairment showed a stronger relationship with outcomes [13]. Furthermore, studies of AE have shown that neither the presence nor the extent of honeycombing predicts short‐term mortality in IPF, suggesting that acute inflammatory injury and diffuse alveolar damage may outweigh the prognostic influence of pre‐existing fibrosis in this setting [14]. The widespread use of AFT in the present cohort may have further reduced any prognostic differences associated with radiological UIP. By slowing fibrotic progression and functional decline, antifibrotic agents may attenuate the adverse natural history traditionally attributed to honeycombing, thereby contributing to convergence of long‐term outcomes between radiological subgroups. Taken together, these findings suggest that although honeycombing remains an important radiological marker of established fibrosis, its prognostic value within contemporary treated IPF cohorts is limited. Prognosis in IPF may therefore be determined more strongly by physiological impairment, disease behavior, and treatment response than by baseline HRCT pattern alone. Beyond radiological and physiological determinants, emerging evidence suggests that biological pathways involved in tissue repair and metabolic regulation, including thyroid hormone signaling, may also contribute to disease progression and remodeling [15]. These observations further support the concept that the clinical course of fibrotic lung disease cannot be fully explained by radiological fibrosis alone and highlight the need for multidimensional approaches to risk stratification.

Several limitations of this study should be acknowledged. First, all patients received AFT, which may have attenuated potential differences in disease progression and survival between patients with and without a radiological UIP pattern. Because antifibrotic agents target key pathways involved in fibrotic progression, the prognostic impact of radiological UIP features, including honeycombing, reported in untreated cohorts may have been diminished in this treated population. Second, radiological classification was based solely on baseline HRCT findings. Longitudinal changes in fibrotic abnormalities, including the development or progression of honeycombing during follow‐up, were not assessed and therefore could not be incorporated into the prognostic analysis. Third, survival was evaluated throughout the entire follow‐up period regardless of treatment discontinuation or completion. Consequently, the duration of survival follow‐up exceeded the duration of AFT in some patients. Furthermore, dose reductions were frequently required because of adverse events or tolerability issues, reflecting real‐world clinical practice. These treatment‐related factors may have influenced long‐term outcomes and introduced heterogeneity in treatment exposure. Finally, the retrospective multicenter design limited the availability of certain clinical variables. In particular, symptom‐related data, such as nonproductive cough and exertional dyspnea, were not systematically collected across participating centers and therefore could not be included in the present analysis.

## 5. Conclusions

In this cohort of patients with IPF uniformly treated with antifibrotic agents, the presence of radiological UIP or honeycombing was not independently associated with long‐term prognosis. Multidimensional risk assessment incorporating physiological impairment and disease behavior may provide more accurate prognostic stratification in treated UIP populations, as yet to be widely assessed using a multidisciplinary‐based approach.

## Funding

No funding was received for this study.

## Ethics Statement

This study followed the guidelines of the Declaration of Helsinki and was approved by the institutional review board (IRB) at Yokohama City University Hospital (approval No. B190300032). Due to the retrospective nature, the enrolled patients provided verbal informed consent to participate in this study. The description of the study was provided to these patients on an opt‐out basis via the Yokohama City University website (https://www.yokohama-cu.ac.jp/amedrc/ethics/ethical/fuzoku_optout.html). In addition, IRB‐approved procedures for the description of this study have been obtained.

## Consent

The authors have nothing to report.

## Conflicts of Interest

The authors declare no conflicts of interest.

## Supporting Information

Additional supporting information can be found online in the Supporting Information section.

## Supporting information


**Supporting Information** The supporting materials include Supporting Figure 1, showing the inverse probability weighting (IPW)‐adjusted Kaplan–Meier survival curve for event‐free survival, and Supporting Tables 1 and 2, presenting the baseline characteristics after IPW adjustment and the IPW‐adjusted Cox proportional hazards analysis, respectively.

## Data Availability

The datasets used or analyzed during the current study are available from the corresponding author on reasonable request.
