# Supplementary material for: Clinical Impact of HRCT UIP Pattern Among Patients With Idiopathic Pulmonary Fibrosis Undergoing Antifibrotic Treatment: A Multicenter Retrospective Observational Study
Source: Can Respir J. 2026 Sep 10;2026:3604892. doi: 10.1155/carj/3604892 (PMC13563058; doi:10.1155/carj/3604892)
Supplement: Supplementary file 1 [file CARJ-2026-3604892-s001.docx]

**Supplementary Figure 1. Inverse probability weighting-adjusted survival curves for event-free survival in patients with UIP and non-UIP patterns**

Inverse probability weighting (IPW)-adjusted Kaplan–Meier survival curves for event-free survival in patients with UIP and non-UIP patterns. Weighting was performed using age, percentage predicted forced vital capacity (%FVC), and percentage predicted diffusing capacity of the lungs for carbon monoxide (%DLCO). Blue and red lines indicate the UIP and non-UIP pattern groups, respectively. No statistically significant difference in event-free survival was observed between the two groups after adjustment.

**Abbreviations:** DLCO, diffusing capacity of the lungs for carbon monoxide; FVC, forced vital capacity; IPW, inverse probability weighting; UIP, usual interstitial pneumonia.

**Supplementary Table 1. Baseline characteristics after inverse probability weighting**

|  | **Non-UIP pattern** | **UIP pattern** | **SMD** |
| --- | --- | --- | --- |
| Raw n | 40 | 97 |  |
| n | 41.28 | 94.73 |  |
| age (mean (SD)) | 73.13 (8.57) | 73.21 (6.60) | 0.01 |
| Sex, male (%) | 33.0 (80.0) | 75.3 (79.5) | 0.01 |
| %FVC (mean (SD)) | 77.74 (15.59) | 78.73 (18.77) | 0.06 |
| %DLCO (mean (SD)) | 61.49 (19.32) | 59.98 (21.79) | 0.07 |
| Nintedanib (%) | 22.8 (55.1) | 42.9 (45.3) | 0.20 |

**Footnote:** Data are presented as weighted median (interquartile range) or weighted number (%). Weighting was performed using age, percentage predicted forced vital capacity (%FVC), and percentage predicted diffusing capacity of the lungs for carbon monoxide (%DLco).

**Abbreviations:** DLco, diffusing capacity of the lungs for carbon monoxide; FVC, forced vital capacity; GAP, gender–age–physiology; HRCT, high-resolution computed tomography; IPW, inverse probability weighting; SMD, standardized mean difference; UIP, usual interstitial pneumonia.

**Supplementary Table 2. Inverse probability weighting-adjusted Cox proportional hazards analysis for event-free survival**

| **Variable** | **HR** | **Lower_95CI** | **Upper_95CI** | **P_value** |
| --- | --- | --- | --- | --- |
| UIP | 1.275831 | 0.687834 | 2.366476 | 0.44 |

**Footnote:** Cox proportional hazards analysis was performed after inverse probability weighting adjustment using age, percentage predicted forced vital capacity (%FVC), and percentage predicted diffusing capacity of the lungs for carbon monoxide (%DLCO) as covariates.

**Abbreviations:** CI, confidence interval; DLCO, diffusing capacity of the lungs for carbon monoxide; HR, hazard ratio; IPW, inverse probability weighting; UIP, usual interstitial pneumonia.
